# Assessment of knowledge, attitudes and practice towards Vitamin D among university students in Pakistan

**DOI:** 10.1186/s12889-020-8453-y

**Published:** 2020-03-18

**Authors:** Amina Tariq, Shanchita R. Khan, Amna Basharat

**Affiliations:** 1 School of Public Health and Social Work, Queensland University of Technology, Kelvin Grove, QLD, Brisbane, 4059 Australia; 2 National University of Computer and Emerging Sciences (NUCES), Islamabad, Pakistan

**Keywords:** Vitamin D, Young adults, Knowledge, Pakistan

## Abstract

**Background:**

Pakistan has one of the highest reported incidence of vitamin D deficiency in studies conducted worldwide. However, there has been very limited exploration of vitamin D related knowledge, attitudes and practices among healthy youth in Pakistan.

**Methods:**

A cross-sectional survey was conducted among youth (aged > 16 years) from two engineering universities in Pakistan. Participants were asked questions on their concern about vitamin D levels, testing, and supplementation practices. Knowledge was examined using questions about food sources, health benefits and factors affecting vitamin D production within the human body. Of the 900 eligible students invited to participate, 505 (56%) completed the questionnaire and were included in the analysis.

**Results:**

Only 9% participants were able to identify the correct food sources of vitamin D, 33% were aware of the bone health benefits (bone health and calcium absorption) of vitamin D and 36% identified sunlight exposure as a factor influencing vitamin D production. Knowledge about food sources and health benefits of vitamin D was not associated with gender and individuals concern about their levels. Those tested and taking supplements were more likely to identify bone related health benefits and factors affecting vitamin D production. Forty percent male and 52% female students expressed concern that their vitamin D levels were too low. However, 72% participants reported that they had never been tested for vitamin D levels. Use of supplements was significantly higher among female students (F = 52% vs M = 37%; *P* = 0.003). Those who had been tested for vitamin D deficiency were more likely to take supplements.

**Conclusion:**

Despite being identified as a high-risk population, knowledge about vitamin D was limited among university students. Interventions are needed to increase awareness about the importance of vitamin D for health, including the need for exposure to sunlight and adequate dietary intake of vitamin D. Our study provides much needed baseline evidence for making health-policy recommendations for this vulnerable population group.

## Background

There is an increasing body of evidence that suggests vitamin D deficiency is becoming a global epidemic, with South Asians being one of the most affected population groups [1–3]. Exposure to sunlight is the main source of vitamin D. Some of the best dietary sources of vitamin D include oily fish and egg yolks, as well as vitamin D dietary supplements [4, 5]. Despite receiving plentiful sunlight throughout the year in their countries, South Asians remain one of the most vulnerable populations to suffer from low vitamin D levels [6]. A number of studies that have measured vitamin D levels among South Asian population groups have indicated a high prevalence of its deficiency among healthy young adults, women and infants [7–9].

Although the biological factors that reduce serum vitamin D levels are known, the knowledge and attitudes towards vitamin D need further investigation especially in dark-skinned population of South Asian countries [10–12]. Ethno-cultural factors, environmental factors, and behavioural practices which limit sun exposure may contribute to vitamin D deficiency in these populations [13–15]. These factors are not well-understood and have not been extensively examined especially in a South Asian context.

Pakistan is a sundrenched South Asian country with cutaneous production of vitamin D possible throughout the year. However, the recent national nutrition survey of Pakistan reported a high prevalence of vitamin D deficiency [16–18]. Studies exploring vitamin D deficiency in Pakistan have identified it to be widespread among various segments of the population including asymptomatic adult population, ambulatory patients, mothers and their newborns [16, 19, 20]. Ninety percent of university students, especially females, aged between 18 and 35 years have been reported as vitamin D deficient [21–23].

Researchers are calling for public health strategies including supplementation to address high levels of vitamin D deficiency [18, 24]. A recent international report into vitamin D deficiency in the developing world recommended improvement in awareness about vitamin D and its impact of health outcomes among adult population. Nevertheless, there is an absence of studies that examine existing knowledge, attitudes and practices about vitamin D among young adults in high risk countries like Pakistan [3]. Understanding the level of knowledge, attitudes and practice about vitamin D is vital to facilitate policy makers in designing interventions appropriate for the context of Pakistan. The aim of this study therefore was to assess vitamin D related knowledge, attitudes and practices (KAP) among university students in Pakistan. In addition, we also examined possible association of KAP with gender and testing status for vitamin D.

## Methods

A quantitative cross-sectional design was used to conduct this research. An anonymous survey was designed to examine knowledge, attitudes and practices towards vitamin D among university students in Pakistan. Eligibility criteria included both male and female adults (> 16 years) living in the Pakistan, enrolled in an undergraduate or postgraduate program in a tertiary education institute.

The instrument was developed based on review of previous studies consisting of validated items across range of dimensions, including the assessment of skin colour type, sun exposure, dietary knowledge about vitamin D etc. [25–27]. The questionnaire consisted of:

1. Demographics: Age, gender, education, skin colour, type of housing and questions about head covering among female participants.
2. Vitamin D knowledge, attitudes and practices: Knowledge about vitamin D was assessed on the food sources of vitamin D, the health benefits of vitamin D and factors that affect vitamin D production and absorption in the body similar to previous studies exploring vitamin D knowledge [25, 27]. Attitudes towards vitamin D were assessed by asking the participants about their level of agreement with statements that are similar to those validated previously [26, 27]. These included (a) “I am concerned that my vitamin D levels may be too low”; (b) “From a health perspective, it is important to get some sun exposure every day”. Responses were measured using a five-point Likert scale. Practices regarding vitamin D included questions around if participants were ever tested for vitamin D deficiency and their supplementation intake (if any). Questions were also asked about sun exposure practices including time spent outdoors and sunscreen usage.

### Survey validation and administration

A pilot-test of the survey was completed with a sample of 14 undergraduate university students and teachers in Pakistan to ensure that the target audience understood what each question was asking, as well as what each response meant. A member of the research team (AB) then discussed ideas with the pilot participants on how to make the survey easier to read and understand. The pilot feedback was used to revise the survey accordingly.

The final anonymous survey, which took 10 min on average to complete, was administered using online *Key Survey* tool. Eligible participants (university students aged 16 years or above currently enrolled in an undergraduate or post graduate course in Pakistan) were approached using the university group email lists of two leading engineering universities in Pakistan. These lists included all 900 undergraduate and postgraduate engineering students in the selected universities where ethics approval was obtained.

Upon opening the survey platform, participants were asked to read and agree to the terms of an online consent form. The survey remained open for a period of eight weeks between November 2017 – January 2018. Following strategies were adopted to minimise potential non-response bias: short survey length (< 10 min) with email reminders sent every two weeks.

### Data analysis

A data matrix was produced from the completed questionnaires using SAS 9.4 for Windows (SAS Institute Inc.). The main outcomes of the study were knowledge about food sources, health benefits and factors affecting vitamin D production; attitude about vitamin D; and vitamin D testing and supplementation practices. Descriptive statistics were used to present demographic data and to evaluate knowledge, attitude and practices towards vitamin D. We summarized data as frequencies (numbers and percentages) for categorical variables. Univariate and bivariate associations between categorical variables (demographics, attitudes, practices and knowledge) were established by chi square (χ^2^) test. Where required, *P* ≤ .05 was considered significant. We followed the Strengthening the Reporting of Observational Studies in Epidemiology (STROBE) guidelines for the presentation and writing of our manuscript.

### Ethics approval

Ethics approval for the study was obtained from QUT Human Research Ethics Committee (Approval number: 1700000897). The approved ethics was ratified by human research ethics committees at National University of Computer and Emerging Sciences (NUCES), Islamabad, Pakistan and International Islamic University, Islamabad, Pakistan.

## Results

Students from universities in Islamabad, Pakistan were invited to participate in the study. Of the 900 eligible students invited to participate, 559 (62%) logged onto the online questionnaire platform. Of these, 505 (56% of 900) completed the questionnaire with full information on sociodemographic characteristics; and, therefore, were included in the final analysis. Fifty-eight percent of participants identified as male and 42% as female. The sample was almost homogeneous in terms of age and education with majority being 16–25 years old (88%) and undergraduate (85%) students. General characteristics of the surveyed population are summarised in Table 1.

**Table 1:** General characteristics of participants

Variables	N (%)
**Gender**	
Male	294 (58.2)
Female	211 (41.8)
**Age group (years)**	
16–25	445 (88.3)
26–30	38 (7.5)
30+	21 (4.2)
**Skin colour**	
Fair or pale	149 (32.3)
Fair to beige	130 (28.2)
Light brown (olive)	160 (74.7)
Dark brown	22 (4.8)
**Education level currently enrolled in**	
Bachelor degree	431 (92.7)
Postgraduate degree	74 (14.7)
**Housing**	
Apartment	93 (18.4)
House (≤3 bedrooms)	176 (34.9)
House (> 3 bedrooms)	212 (42.0)
Hostel	21 (4.2)
Other	3 (0.6)
**Marital status**	
Single	468 (92.7)
Married/widowed	37 (7.3)
**Women regularly wear covering that covers face/head**	
Yes	169 (80.1)
No	42 (19.9)

### Vitamin D – knowledge of food sources, health benefits and factors affecting production

When given a list of food items and asked to identify the correct sources of vitamin D, only 9% male and 13% female students were able to identify the correct sources of vitamin D (Table 2). Of the 505 students surveyed, 51% identified fruits and 41% identified vegetables as the possible food sources of vitamin D. Only 27% students identified egg yolks, 33% fish and 29% fish oil as the correct food sources. Knowledge about food sources was not associated with gender. Those who had been tested, reported slightly higher knowledge of food sources when compared to those who have never been tested (*P* = .043; Table 3).

**Table 2:** Association of vitamin D knowledge, attitudes and practices with gender

Variables	N (%)		***P*** value
	Male	Female	
**Related to Knowledge about Vitamin D**			
Knowledge about food sources			
Only correct	22 (8.5)	24 (13.1)	.1139
Incorrect with/without correct	238 (91.5)	159 (86.9)	
Missing: 62			
Knowledge about health benefits			<.0001
Bone benefits +/− other benefits	160 (62.3)	152 (82.0)	
Other benefits only	53 (20.6)	16 (8.7)	
Don’t know	44 (17.1)	17 (9.3)	
Missing: 65			
Knowledge about factors affecting Vitamin D			.0943
Sun exposure and UV index with/without other factors	117 (46.1)	98 (54.4)	
Only other factors	91 (35.8)	47 (26.1)	
Don’t know	46 (18.1)	35 (19.4)	
Missing: 71			
**Related to Attitudes towards Vitamin D**			
Concerned current vitamin D levels may be too low			
Agree/strongly agree	95 (40.4)	85 (51.8)	.05
Neutral	92 (39.2)	57 (34.8)	
Disagree/strongly disagree	48 (20.4)	22 (13.4)	
Missing: 106			
From a health perspective, it is important to get some sun exposure every day			.103
Agree/strongly agree	177 (73.4)	144 (82.3)	
Neutral	46 (19.1)	23 (13.1)	
Disagree/strongly disagree	18 (7.5)	8 (4.6)	
Missing: 89			
**Related to Practices towards Vitamin D**			
Vitamin D testing			.819
Never tested	191 (72.4)	132 (73.3)	
Ever tested	73 (27.7)	48 (26.7)	
Missing: 61			
Ever take supplements or multivitamin containing vitamin D			.003
No	152 (63.3)	84 (48.6)	
Yes	88 (36.7)	89 (51.5)	
Missing: 92			
Vitamin D dose of supplement			.002
< 1000 IU	48 (54.6)	24 (28.2)	
1000 IU and over	5 (5.7)	8 (9.4)	
Don’t know	35 (39.8)	53 (62.4)	
Missing: 173			
Motivation for starting to take vitamin D			.083
A doctor or health professional recommended	31 (35.2)	40 (47.1)	
A friend or family member recommended	14 (15.9)	16 (18.8)	
Read about vitamin D on the internet or in a book/magazine	25 (28.4)	11 (12.9)	
Someone else in the family is taking it	18 (20.5)	18 (21.2)	
Missing: 338			
Primary reason for not taking vitamin D			.305
Never thought about it	94 (62.3)	62 (73.8)	
Feel that get enough vitamin D from my diet	18 (11.9)	8 (9.5)	
Feel that get enough vitamin D from sun	19 (12.6)	8 (9.5)	
Prefer not to take any supplements/vitamins	20 (13.3)	6 (7.1)	
Missing: 270			
Time spent outdoors on a typical workday			
15 min or less	39 (14.6)	40 (21.1)	.136
16 min - < 1 h	120 (44.9)	72 (37.9)	
1 h or more	108 (40.5)	78 (41.1)	
Missing: 48			
Time spent outdoors on a typical weekend day			<.0001
15 min or less	43 (16.0)	75 (39.5)	
16 min - < 1 h	115 (42.9)	68 (35.8)	
1 h or more	110 (41.0)	47 (24.7)	
Missing: 47			
Sunscreen use			.196
No	153 (56.9)	97 (50.8)	
Yes	116 (43.1)	94 (49.2)	
Missing: 45

**Table 3:** Association of vitamin D knowledge, attitudes and practices with vitamin D testing

Variables	N (%)		***P*** value
	Never tested	Ever tested	
**Related to Knowledge about Vitamin D**			
Knowledge about food sources			.0433
Only correct	27 (8.5)	18 (15.1)	
Incorrect with/without correct	290 (91.5)	101 (84.9)	
Missing: 62			
Knowledge about health benefits			<.0001
Bone benefits +/− other benefits	227 (72.3)	76 (63.9)	
Other benefits only	31 (9.9)	38 (31.9)	
Don’t know	56 (17.8)	5 (4.2)	
Missing: 65			
Knowledge about factors affecting Vitamin D			<.0001
Sun exposure and UV index with/without other factors	165 (52.7)	45 (39.5)	
Only other factors	76 (24.3)	60 (52.6)	
Don’t know	72 (23.0)	9 (7.9)	
Missing: 71			
**Related to Attitudes towards Vitamin D**			
Concerned current vitamin D levels may be too low			.168
Agree/strongly agree	118 (41.2)	58 (52.3)	
Neutral	113 (40.2)	35 (31.5)	
Disagree/strongly disagree	50 (17.8)	18 (16.2)	
Missing: 113			
From a health perspective, it is important to get some sun exposure every day			.981
Agree/strongly agree	231 (77.3)	84 (76.4)	
Neutral	50 (16.7)	19 (17.3)	
Disagree/strongly disagree	18 (6.0)	7 (6.4)	
Missing: 96			
**Related to Practices towards Vitamin D**			
Ever take supplements or multivitamin containing vitamin D			<.0001
No	203 (71.7)	31 (26.3)	
Yes	80 (28.3)	87 (73.7)	
Missing: 104			
Vitamin D dose of supplement			.0001
< 1000 IU	22 (27.5)	49 (56.3)	
1000 IU and over	4 (5.0)	8 (9.2)	
Don’t know	54 (67.5)	30 (34.5)	
Missing: 338			
Motivation for starting to take vitamin D			.03
A doctor or health professional recommended	34 (42.5)	33 (37.9)	
A friend or family member recommended	13 (16.3)	17 (19.5)	
Read about vitamin D on the internet or in a book/magazine	11 (13.8)	25 (28.7)	
Someone else in the family is taking it	22 (27.5)	12 (13.8)	
Missing: 338			
Primary reason for not taking vitamin D			.0005
Never thought about it	143 (70.4)	13 (41.9)	
Feel that get enough vitamin D from my diet	16 (7.9)	10 (32.3)	
Feel that get enough vitamin D from sun	22 (10.8)	4 (12.9)	
Prefer not to take any supplements/vitamins	22 (10.8)	4 (12.9)	
Missing: 271			
Time spent outdoors on a typical workday			.4
15 min or less	47 (14.7)	24 (19.8)	
16 min - < 1 h	141 (44.1)	48 (39.7)	
1 h or more	132 (41.3)	49 (40.5)	
Missing: 64			
Time spent outdoors on a typical weekend day			.85
15 min or less	80 (24.8)	31 (25.6)	
16 min - < 1 h	129 (40.1)	51 (42.2)	
1 h or more	113 (35.1)	39 (32.2)	
Missing: 62			
Sunscreen use			<.0001
No	201 (62.2)	43 (35.5)	
Yes	122 (37.8)	78 (64.5)	
Missing: 61			

When asked about the health benefits of vitamin D, 17% males and 9% females reported that they did not know (Table 2). Gender was associated with identifying health benefits, with females more likely to report bone related health benefits (*P* < .0001). Seventeen percent of those who had never been tested reported that they did not know about any health benefits of vitamin D, while only 4% of those who were tested reported lack of knowledge of benefits of vitamin D (Table 3). Supplementation practices were also associated with the health benefit aspect of vitamin D knowledge, with those taking supplement more likely to be aware of bone related health benefits (*P* = .0002; Supplementary Table 1).

Knowledge about factors that influence body’s ability to make vitamin D was poor with 1 in 5 participants reporting that they did not know which factors influenced vitamin D production. Only 36% participants identified that sunlight exposure influenced vitamin D production. About 33% participants thought age had an influence, while 22% thought skin colour determined vitamin D production in the body. Only 18% participants identified UV index and 13% identified clothing as factors influencing vitamin D levels in the body. Gender was not associated with knowledge about factors affecting vitamin D production (Table 2). Twenty three percent of those who had never been tested reported that they did not know about factors affecting vitamin D production (*P* < .0001; Table 3). Supplementation status was associated with knowledge about factors affecting vitamin D production (*P* = .0039; Supplement 1).

### Vitamin D – attitudes, testing, supplementation and sun exposure practices

When asked if concerned about their current vitamin D levels being too low, 40% of male students and 52% female students agreed that they were concerned their levels were too low (Table 2). However, despite the widespread concern, overall 72% of participants reported that they had never been tested for vitamin D levels. Forty-one percent of those who expressed concern about their vitamin D levels being too low had never been tested.

More than half of the students (52%) had never taken any supplements containing vitamin D. Vitamin D supplement use was significantly higher among female students (F = 52% vs M = 37%; *P* = .003). Nevertheless, a higher proportion of female students were unaware of the dose of vitamin D they were taking (F = 62% vs M = 40%; *P* = .002). More than half of the males (55%), who were taking vitamin D supplements reported taking less than 1000 IU of the vitamin and only 5% of those who took supplements, reported taking 1000 IU or more of the vitamin (Table 2). About 32% of the students who took vitamin D supplements took them all year round, while about 35% took them for the time prescribed by the doctor. Despite reporting concern for their vitamin D levels, most students reported not taking vitamin D because they had never thought about it (F = 74% vs M = 62%). Only 10% female and 13% male students thought they got enough vitamin D through sun exposure.

Testing for vitamin D deficiency was associated with supplementation practices with 74% of those who had reported being tested taking supplement containing vitamin D (*P* < .0001). However, attitudes towards vitamin D and perceived sun exposure need were not associated with the testing status (Table 3).

More than 40% of the participants reported spending an hour or more outdoors between 8 am and 4 pm on a workday, while around 17% spent 15 min or less outdoors during those hours. There was no significant difference among male and female students’ time spent outdoors during the weekdays. However, the amount of time spent outdoors between 8 am and 4 pm on a weekend day differed significantly among male and female students (Table 2) with 41% males and 25% female students spending an hour or more outdoors on a weekend day (*P* < .0001).

Half of the participants reported never using sunscreen. Those who did use sunscreen, mostly used it on their face and hands. Eight in ten female participants reportedly wore head covering, while 32% of those, wore clothing that covered their faces as well (Table 1). Only 33% of the males ever wore caps or hats. Use of sunglasses was also low with 62% males and 76% females rarely or never wearing them. The majority reported hot weather (57%) to be the most common reason, while indoor lifestyle (31%) and cosmetic reasons (29%) were also listed as common reasons for avoiding sun exposure.

## Discussion

Our study is one of the very few which aim at understanding at knowledge, attitudes and practices towards vitamin D among healthy university students in Pakistan, a country marked by very high deficiency of vitamin D across the entire population [24].

Despite this cohort being university students and having access to the internet and health information, knowledge about vitamin D was quite poor. Poor knowledge could be due to conflicting information available about vitamin D and lack of clear localised messages about sun exposure. Similar limitations to the knowledge of vitamin D have been reported in studies across Bangladesh, the Middle East, Iran, Lebanon and also among immigrants from South Asian countries living across Europe. Similar to our study, most studies have found their participants not able to identify sources of vitamin D in food and sunlight as critical enabler in vitamin D production [28, 29]. However, our results are in contrast to those reported in the UK where the participants did demonstrate good level of knowledge about vitamin D [30]. This perhaps is indicative of the increased public awareness and food fortification practices in the developed world. Multi-prong strategies are required to appraise public knowledge and awareness about vitamin D. People should receive information, in local languages, that reflects the actual state of knowledge regarding vitamin D and its association with health, along with clear information on vitamin D sources [31]. These national public health messages should be relayed across various platforms (i.e., social media, health centres, universities, schools), especially through the media, to increase awareness among all sub-groups of the population [31]. Further it is important to recognise awareness regarding the causes and prevention of vitamin D deficiency as an avenue to be explored by the public health offices, which should begin by conducting conclusive studies to determine general public knowledge regarding this pertinent issue [24]. Food Fortification Programme (FFP) in Pakistan is exploring the food fortification of staple foods like milk, vegetable oil and wheat with vitamin D [32, 33]. This is in alignment with evidence that suggests universal vitamin D food fortification can improve serum 25-hydroxyvitamin D levels [34, 35]. However, to encourage public adaptation, it is important to address any public concerns about food fortification, which have been reported to exist especially among South Asians [12, 36]. In addition, an effective strategy to increase awareness may be to conduct awareness campaigns which offer public funded vitamin D level testing for high risk communities.

To our knowledge this is the first study that examined testing and supplementation practices in relevance to vitamin D among young adults in Pakistan. Despite widespread concern about vitamin D levels being low, majority of participants (72%) in our study had never been tested for vitamin D levels. Vitamin D tests are costly in Pakistan and act as a deterrent for people getting tested to check their vitamin D levels [37]. This may indicate why educated young adults would not get tested for vitamin D levels despite their concerns. Getting tested, however, was related to use of supplementation. This is in line with Agens et al. who reported that physicians who had patients vitamin D tested were linked with recommending higher dose of supplement to patients [38]. Therefore, reducing the testing costs to enable timely testing would help in early identification of the vitamin D deficiency. To reduce testing costs, there is an increasing call for developing diagnostic kits locally in Pakistan [39].

Fifty-two percent of participants in our study were not taking any supplements, but among those who reported taking them, females were the more likely users of supplements. This finding may be indicative of prevalent discussions in mainstream media channels that particularly target young women to have adequate vitamin D in early 20s and encourage use of supplements [24, 40]. We recommend extension of these discussions to the general population including the other population groups of the society. It is important to note that low doses were reported by those taking supplements irrespective of the gender with more than half not knowing the dose they were taking. To address the severe deficiency cases, clinicians are recommending higher dose supplementation especially in context of Pakistani population [41]. Although there is a need to timely identify and treat severe vitamin D deficiency, such medicalised view towards vitamin D may highlight the existing culture of testing and treating with tablets among South Asians [12]. This approach may only help in short–term and can be ineffective for addressing this concerning problem for the long term [15, 29, 31, 42, 43].

In terms of sun exposure practices, both male and female participants reported spending similar amount of time outdoors during weekdays. However, time spent outdoors on weekend days differed significantly between males and females. This finding corroborates with studies that have exclusively examined the vitamin D status and reasons for its deficiency among women in Pakistan, and cited lifestyle, the clothing, season, and outdoor activities as primary contributing factors. In our study, 80% of females reported wearing clothing that covers their heads when outdoors, reflective of the Muslim majority population of the country. However, similar to other studies our results did not show vitamin D knowledge, attitudes or practices to be associated with veiling [44, 45]. Therefore, irrespective of the veiling practices, female participants spent less time outdoors on weekends. This perhaps is reflective of the conservative culture and lack of safe outdoor spaces exclusively for women. To address the magnitude of the problem, vitamin D deficiency should be treated like a public health epidemic at national level and introduce public awareness programs like that for iodine deficiency which have been successful in the Pakistani context [46].

## Limitations

The study has several limitations first of which is it was done on a convenience sample and selection bias might be present since the study was carried out in a specific university setting. Hence the results cannot be generalized for the Pakistani population. The limited age group range also adds to the limitations of this study. Future research should validate this questionnaire so that it can be used in a nationwide larger study. Importantly, serum 25-hydroxyvitamin D should be collected so that association between knowledge, attitudes and practices with vitamin D levels can be tested. In addition, further studies should explore ways in which innovative information channels like social media can improve public awareness and their impact using diffusion of innovation framework [47].

## Conclusion

The majority of the study participants lacked in knowledge about vitamin D and had inconsistent practices towards managing its deficiency. Despite widespread concern about vitamin D levels being low, testing for vitamin D was not common in our population. Further research, on a larger scale, is needed in this area to enable a better understanding of the knowledge and attitudes about vitamin D in high risk South Asian populations and how interventions like fortified foods or sun exposure advices should be implemented for their long-term effectiveness.

## Supplementary information

**Additional file 1: Supplement 1.** Association of vitamin D supplement intake with knowledge. **Supplement 2.** Association of concern about vitamin D intake with knowledge.

## Data Availability

The datasets during and/or analysed during the current study are available from the corresponding author on reasonable request.

## Abbreviations

M: Male
F: Female
