# Supplementary material for: Assessment of knowledge, attitudes and practice towards Vitamin D among university students in Pakistan
Source: BMC Public Health. 2020 Mar 18;20:355. doi: 10.1186/s12889-020-8453-y (PMC7079348; doi:10.1186/s12889-020-8453-y)
Supplement: Supplementary file 1 — Additional file 1: Supplement 1. Association of vitamin D supplement intake with knowledge. Supplement 2. Association of concern about vitamin D intake with knowledge. [file 12889_2020_8453_MOESM1_ESM.docx]

**Supplement 1: Association of vitamin D supplement intake with knowledge**

| **Variables** | **Number (%)** | | ***P* value** |
| --- | --- | --- | --- |
|  | **Ever take vitamin D supplements** | |  |
|  | No | Yes |  |
| Knowledge about food sources  Only correct  Incorrect with/without correct  Missing: 104 | 20 (8.7)  209 (91.3) | 22 (12.8)  150 (87.2) | 0.189 |
| Knowledge about health benefits  Bone benefits +/- other benefits  Other benefits only  Don’t know  Missing: 106 | 156 (68.4)  31 (13.6)  41 (18.0) | 129 (75.4)  34 (19.9)  8 (4.7) | 0.0002 |
| Knowledge about factors affecting Vitamin D  Sun exposure and UV index with/without other factors  Only other factors  Don’t know  Missing: 113 | 105 (46.7)  68 (30.2)  52 (23.1) | 89 (53.3)  61 (36.5)  17 (10.2) | 0.0039 |

**Supplement 2: Association of concern about vitamin D intake with knowledge**

| **Variables** | **Number (%)** | | | ***P* value** |
| --- | --- | --- | --- | --- |
|  | **Concerned current vitamin D levels may be too low** | | |  |
|  | Agree/  strongly agree | Neutral | Disagree/  strongly disagree |  |
| Knowledge about food sources  Only correct  Incorrect with/without correct  Missing: 106 | 16 (8.9)  164 (91.1) | 14 (9.4)  135 (90.6) | 9 (12.9)  61 (87.1) | 0.626 |
| Knowledge about health benefits  Bone benefits +/- other benefits  Other benefits only  Don’t know  Missing: 108 | 132 (73.7)  32 (17.9)  15 (8.4) | 113 (76.4)  17 (11.5)  18 (12.2) | 47 (67.1)  10 (14.3)  13 (18.6) | 0.117 |
| Knowledge about factors affecting Vitamin D  Sun exposure and UV index  with/without other factors  Only other factors  Don’t know  Missing: 113 | 82 (45.6)  63 (35.0)  35 (19.4) | 83 (55.7)  44 (29.5)  22 (14.8) | 35 (50.0)  24 (34.3)  11 (15.7) | 0.462 |
